# Mesenchymal Stem Cells for Coronavirus (COVID-19)-Induced Pneumonia: Revisiting the Paracrine Hypothesis with New Hopes?

**DOI:** 10.14336/AD.2020.0403

**Published:** 2020-04-02

**Authors:** Selçuk Öztürk, Ayşe Eser Elçin, Yaşar Murat Elçin

**Affiliations:** Tissue Engineering, Biomaterials and Nanobiotechnology Laboratory, Faculty of Science, Ankara University, Ankara, Turkey

**Keywords:** COVID-19, mesenchymal stem cells, cell transplantation, pneumonia, immunomodulation

## Abstract

Mesenchymal stem cells (MSCs) bear a promising potential for regenerative medicine therapies and they repair damaged tissue through secretion of immune modulatory and anti-inflammatory molecules acting in a paracrine fashion. Coronavirus disease 2019 (COVID-19) has spread all over the world with high morbidity and mortality rates and there is no specific treatment for this infection. A recent study published in the journal reports that MSC infusion is safe and effective in patients suffering from COVID-19 induced pneumonia. In the light of this study and previous reports, we make additional comments about possible therapeutic effects of MSCs in COVID-19 infection.

Since the first discovery of mesenchymal stem cells (MSCs) by Friedenstein and colleagues in 1970s [1], their regenerative and therapeutic potential has been widely investigated in several diseases with promising results [2]. Initially, the principal mechanism underlying their therapeutic effects was thought to be transdifferentiation and/or fusion of MSCs into the damaged tissue. However, their poor survival, low engraftment and differentiation capacity in the damaged tissue has refuted the idea that administrated MSCs repair tissue damage through replacing dead cells with newly differentiated and functional cells. Subsequent studies have shown that these cells secrete bioactive molecules such as growth factors, chemokines, cytokines and extracellular vesicles acting in a paracrine fashion and exert immune modulation and anti-inflammatory effects in tissue of interest [3]. Accordingly, the mechanism namely “paracrine hypothesis” has emerged as an alternative explanation in recent years for the beneficial effects of MSCs observed in preclinical and clinical studies [3, 4].

The world has encountered a new pandemic named as novel Coronavirus disease 2019 (COVID-19) since December 2019 which started from China and spread all over the world in a few months. Clinical situation of COVID-19 infection varies from mild fever to respiratory failure resulting with acute respiratory distress syndrome (ARDS) and death. Increased proinflammatory cytokine activation with detrimental alterations in the lungs have been suggested as hallmark in the pathogenesis of infection. Since there is no effective cure or vaccine, primary prevention strategies such as avoiding close contact and attention to personal hygiene are strongly recommended. However, the number of patients suffering from COVID-19 infection is increasing utmost and some of them need hospitalization and/or intensive care unit (ICU) follow-up [5-7]. Therefore, finding an effective cure for patients suffering from the COVID-19 infection is urgently needed.

In the last issue of the journal, Leng et al. [8] published the results of seven patients suffering from COVID-19 infection with varying clinical situations and treated with clinical grade human MSCs. The patients received 1 million MSCs per kilogram intravenously and were closely followed for 14 days. The authors of the study reported that there were no adverse effects related with cell transplantation. Besides, the pulmonary functions and symptoms of the patients treated with MSC transplantation improved quickly and three of them were discharged from hospital in ten days as indicated by the authors. Evidence of inflammatory system activation in peripheral blood such as C-reactive protein levels, tumor necrosis factor-alpha levels and cytokine secreting immune system cells were found to be decreased after treatment. Besides, the number of peripheral lymphocytes, dendritic cells, and levels of anti-inflammatory protein interleukin-10 increased suggesting a beneficial effect of MSCs through immune modulation and anti-inflammatory activity in cell treated patients. Moreover, RNA sequencing and gene expression analyses of MSCs revealed high anti-inflammatory and trophic factor activity and cells were free from COVID-19 infection according to the report of the authors [8]. We believe that there are several take home messages which might be gathered from this study and make additional comments about possible therapeutic effects of MSCs in COVID-19 infection.

ARDS, which is a highly complex disease process involving multiple organs, represents the most severe clinical situation of COVID-19 infection and is the major cause of death in ICUs. Our understanding about the therapeutic role of MSCs in ARDS mostly originates from animal model studies suggesting a repair process through modulation of immune system cells and inflammatory/ anti-inflammatory bioactive molecules [9]. Clinical trials testing the safety of MSC infusion in ARDS patients demonstrated no adverse effects of cell therapy [10,11], while the beneficial effects were limited [10]. A recent case presentation also reported successful management of a critically ill COVID-19 patient with human umbilical cord MSC infusion [12]. Likewise, MSC infusion was reported to be safe and effective in the study of Leng and colleagues [8]. Although these reports are promising and encouraging for further studies and clinical applications of MSCs in COVID-19 patients, we believe that these striking results must be interpreted cautiously. Lack of statistical power, randomization, dose studies and long-term follow-up stand out as significant weaknesses of the study. In addition, the number of patients involved in the study is relatively low. Among 7 patients treated with MSC infusion, only one patient was reported to be in critically severe type condition and the other patients were in relatively better clinical condition. Besides, these results need to be confirmed in multicenter trials involving different regions and countries. While commenting about this study, we are also aware that this is a preliminary study and there is an urgency to find an effective treatment for these patients due to the fact that pandemic has spread all over the world and morbidity/mortality rates are sharply increasing.

**Table 1:** Clinical trials recorded in www.clinicaltrials.gov database about stem cells and COVID-19 infection by 27^th^ of March 2020.

Identifier	Phase	Status	Location	Cell type	Estimated enrollment	Primary outcome measure
NCT04252118	1	Recruiting	China	MSC	20	- Size of lesion area by chest radiograph or CT - Side effects in MSC treatment group
NCT04276987	1	Not yet recruiting	China	MSC-derived exosomes	30	- Adverse and severe adverse reaction - Time to clinical improvement
NCT04299152	2	Not yet recruiting	N/A	SCE-treated MNC	20	- Number of patients unable to complete SCE therapy
NCT04273646	N/A	Not yet recruiting	China	UC-MSC	48	- Pneumonia severity index - Oxygenation index
NCT04288102	1-2	Recruiting	China	MSC	90	- Size of lesion area and severity of pulmonary fibrosis by CT
NCT04302519	1	Not yet recruiting	N/A	Dental pulp MSC	24	- Disappear time of ground-glass shadow in the lungs
NCT04313322	1	Recruiting	Jordan	WJ-MSC	5	- Clinical outcome - CT scan - RT-PCR results
NCT04293692	N/A	Withdrawn	China	UC-MSC	N/A	- Size of lesion area by chest imaging - Blood oxygen saturation
NCT04269525	2	Recruiting	China	UC-MSC	10	- Oxygenation index

CT: computerized tomography; MNC: mononuclear cell; MSC: mesenchymal stem cell; N/A: not available; RT-PCR: real-time polymerase chain reaction; SCE: stem cell educator; UC-MSC: umbilical cord mesenchymal stem cell; WJ-MSC: wharton’s jelly mesenchymal stem cell.

From a theoretical perspective, it is reasonable to expect good outcomes from MSCs in COVID-19 infection due to their close intersections regarding pathogenesis of the disease and mechanism of action of MSCs. There are already 9 clinical trials recorded in www.clinicaltrials.gov by 27th of March 2020 aiming to investigate the effect of stem cells in COVID-19 infection which are briefly summarized in Table 1. The results of these studies are eagerly awaited and will shed light on our understanding about the therapeutic role of stem cells to combat with COVID-19 infection. We hope and believe that mankind will find an effective cure to treat this pandemic in the light of science.
